# Generation of Circularly Permuted Fluorescent-Protein-Based Indicators for In Vitro and In Vivo Detection of Citrate

**DOI:** 10.1371/journal.pone.0064597

**Published:** 2013-05-22

**Authors:** Yuki Honda, Kohtaro Kirimura

**Affiliations:** Department of Applied Chemistry, Faculty of Science and Engineering, Waseda University, Tokyo, Japan; Oak Ridge National Laboratory, United States of America

## Abstract

Indicators for citrate, particularly those applicable to its *in vivo* detection and quantitation, have attracted much interest in both biochemical studies and industrial applications since citrate is a key metabolic intermediate playing important roles in living cells. We generated novel fluorescence indicators for citrate by fusing the circularly permuted fluorescent protein (cpFP) and the periplasmic domain of the bacterial histidine kinase CitA, which can bind to citrate with high specificity. The ratiometric fluorescent signal change was observed with one of these cpFP-based indicators, named CF98: upon addition of citrate, the excitation peak at 504 nm increased proportionally to the decrease in the peak at 413 nm, suitable for build-in quantitative estimation of the binding compound. We confirmed that CF98 can be used for detecting citrate *in vitro* at millimolar levels in the range of 0.1 to 50 mM with high selectivity; even in the presence of other organic acids such as isocitrate and malate, the fluorescence intensity of CF98 remains unaffected. We finally demonstrated the *in vivo* applicability of CF98 to estimation of the intracellular citrate concentration in *Escherichia coli* co-expressing the genes encoding CF98 and the citrate carrier CitT. The novel indicator CF98 can be a specific and simple detection tool for citrate *in vitro* and a non-invasive tool for real-time estimation of intracellular concentrations of the compound *in vivo*.

## Introduction

Citrate is a key metabolic intermediate playing important roles in living cells since it is the biomolecule located at the starting point of the tricarboxylic acid (TCA) cycle, also called citric acid cycle [1]. It is also of industrial importance: it has been used for a long time as an acidulant in the manufacture of soft drinks and in the confectionery industry, as a complexing agent in metal treatment, as a monomer for functional and/or biodegradable polymers, and as a water softener in detergents, on the basis of its multimodal properties as an organic acid, chelating agent, and buffering substance [2]. Commercial production of citrate is based on fermentation using the filamentous fungus *Aspergillus niger* and the global production of citrate has increased to approximately 1,750,000 tons in 2011 [3]. The mechanism and machinery of citric acid fermentation by *A. niger* has been studied from the viewpoints of bioscience and biotechnology [2]. In biochemistry, citrate is not only a molecule involved in catabolic processes but also a biosynthetic precursor that exhibits regulatory functions for fatty acid synthesis [4]. In addition, since estimation of citrate concentration in urine is of considerable value in diagnosis of certain diseases, such as kidney stones [5] and prostate cancer [6], qualitative and quantitative analysis of citrate is also of importance in medical field. Hence, indicators applicable to both simple and rapid *in vitro* and *in vivo* detection of citrate have attracted much interest in basic as wells as applied researches.

A number of genetically encoded fluorescent indicators for *in vivo* metabolites have been developed from green fluorescent protein (GFP) and its variants (reviewed in [7]). We also have adopted the visual analysis method using fluorescent protein in studies of citrate production by *A. niger* [8], [9], [10]. The major approach to generating these genetically encoded fluorescent indicators is based on fluorescence resonance energy transfer (FRET), and so far many FRET-based indicators for metabolites have been developed (reviewed in [11], e.g., for cAMP [12], ATP [13], glutamate [14], maltose [15], glucose [16], and citrate [17]). On the other hand, some genetically encoded fluorescent indicators employ an alternative approach based on circularly permuted fluorescent proteins (cpFPs), in which the original N and C termini are connected via a peptide linker, and new N and C termini are created in close proximity to the chromophore [18]. Circularly permutation thus allows placing the fusion of a sensor domain in close proximity to the chromophore environment, which can lead to direct changes in the fluorescence signal upon structural rearrangements initiated by the sensor domain. The calcium indicator Pericam [19], the hydrogen peroxide indicator HyPer [20], [21], and the ATP∶ADP ratio indicator Perceval [22] have been generated by fusing the N and C termini of a cpFP to a specific detector protein for Ca^2+^, hydrogen peroxide, and ATP/ADP, respectively. Conformational changes of fusion proteins caused by the binding of the intended compounds to the detector protein domain lead to fluorescence changes. Therefore, the cpFP system provides us with novel non-invasive fluorescent indicators useful for qualitative and quantitative detection of the binding compounds as well as imaging of localization and dynamics of the fusion proteins. However, to our knowledge, so far there have been no reports concerning cpFP-based indicators for organic acids, including citrate.

In this study, for the first time, we generated a series of novel citrate indicators in which each of the cpFP mutants tested was inserted into the region of *Klebsiella pneumoniae* CitA protein, a highly specific citrate receptor [23], [24], [25], [26], [27], [28], that would undergo great conformational changes upon binding to citrate, examined their properties, and selected the best candidate for the simple and rapid *in vitro* analysis of citrate. Here, we report the molecular and physicochemical properties of the newly generated cpFP-based indicator for citrate and demonstrate its high selectivity against the compound among various organic acids. Finally, its applicability to estimation of *in vivo* citrate concentration in *Escherichia coli* was clearly shown, and the methodology to correct for the intracellular pH effects on its fluorescence signal was developed based on the ratiometric property observed in the excitation spectra of the indicator protein.

## Results

### Generation of cpFP-based indicators for citrate

**Figure 1:**
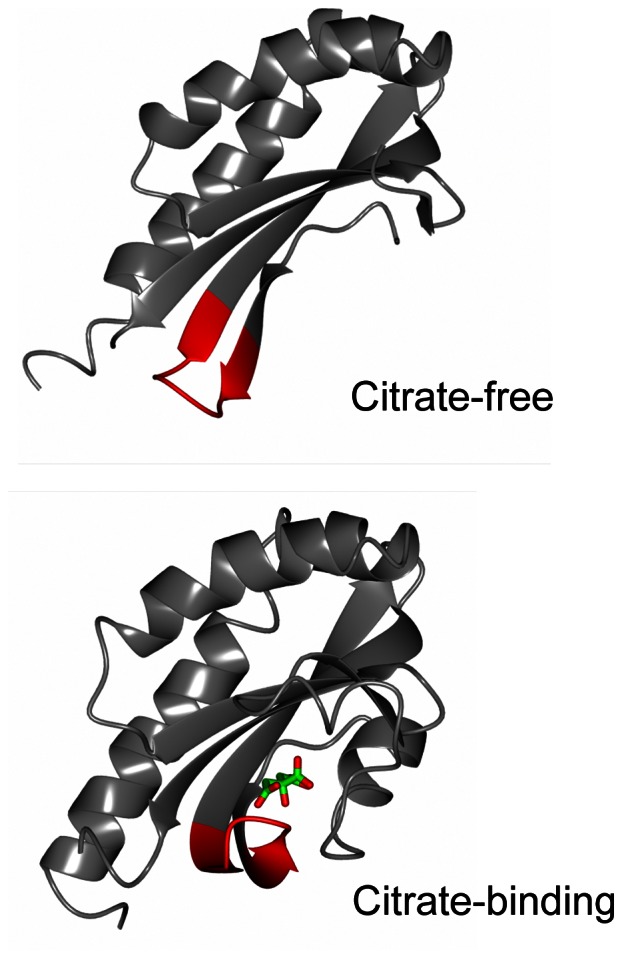
Conformational change of CitAP upon citrate binding. Ribbon representations of citrate-free (2V9A) and citrate-binding (2J80) structures of the periplasmic domain of sensor histidine kinase CitA (CitAP, residues 45–176) are drawn using molecular-graphics software, CCP4mg, based on Protein Data Bank files. Citrate is indicated in stick form (green and red). The red colored ribbon parts (residues 97–105) represent the flexible and target region for the insertion of a circularly permuted fluorescent protein to generate the fluorescent-protein-based indicators for citrate.

To generate a cpFP-based indicator for citrate, we chose the periplasmic domain of the sensor histidine kinase CitA from *K. pneumoniae* [23], [24], [25], [26], [27], [28] as a suitable citrate-binding detector protein on the basis of the following findings: 1) citrate binds to the recombinant sensor domain of CitA (CitAP; corresponding to residues 45–176 of CitA) with high specificity [25], [26], and 2) citrate binding to CitAP results in a dramatic conformational change demonstrated by X-ray and NMR analyses [28], as shown in Figure 1. These ribbon representation of citrate-free and citrate-binding structures of CitAP are drawn using molecular-graphics software CCP4mg [29] based on Protein Data Bank files (citrate free, 2V9A; citrate binding, 2J80). CitA regulates several citrate metabolism genes under anoxic conditions in the presence of environmental citrate in *K. pneumoniae* [23], [24], [25]. Citrate binding to the periplasmic domain of CitA constitutes the trigger for subsequent transmembrane signaling events. In addition, CitA was used as sensor domain of a previously reported FRET-based citrate indicator [17].

In CitAP, the flexible region allowing the insertion of cpFP moiety is located at residues 97–105 (locations of the amino acid residues of CitAP correspond to those of CitA in this paper) according to its structural data [28]. We generated nine chimeric proteins by inserting cpFP into the flexible region of CitAP. The resultant chimeric proteins have the following structures: CitA residues 45-*N*-SAG-cpFP-GT-CitAP residues (*N*+1)-176-His_6_, where *N* is the location number of any one of CitA residues between 97 and 105, and SAG and GT are short amino acid linkers between CitAP and cpFP. These nine chimeric proteins named CF97 to CF105 according to the location number *N* were produced with a hexa-histidine tag (His tag) at C termini using *E. coli* BL21(DE3), and purified by Ni^2+^-affinity chromatography. All of these proteins were confirmed to have a predicted molecular weight of 43.0 kDa by SDS-PAGE. Chimeric proteins CF97, CF98, CF99, CF100, CF101, CF102, and CF103 were obtained in high yields (>1.0 mg-protein/ml), while CF104 and CF105 in low yields (<10 µg-protein/ml).

**Figure 2:**
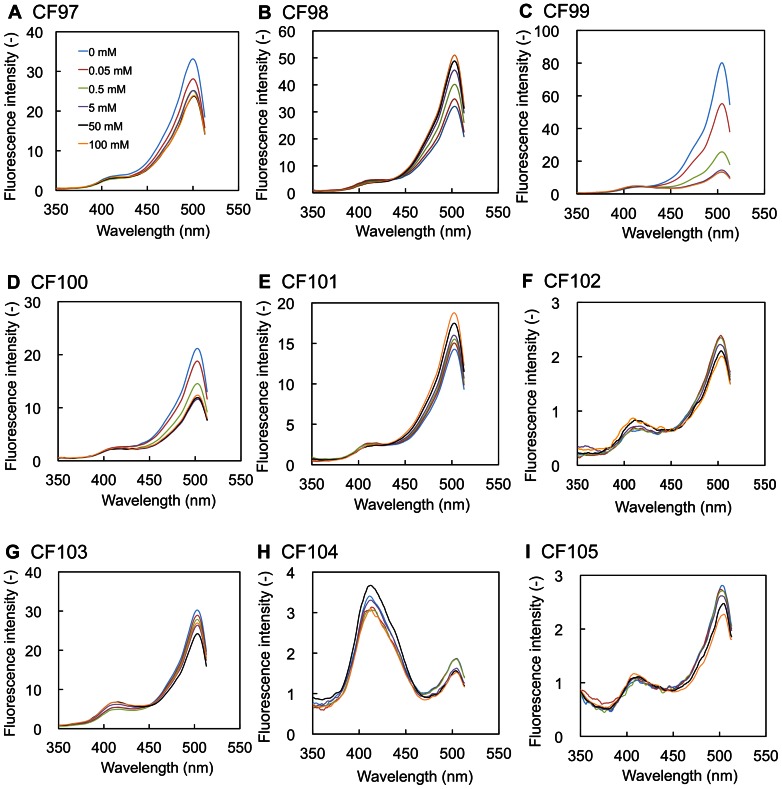
Excitation spectra of nine chimeric proteins, CF97-CF105. Nine chimeric proteins were produced and purified as described in Materials and Methods. The excitation spectra of these chimeric proteins were measured upon the addition of the indicated citrate concentrations. The concentrations of the chimeric protein solutions of CF97, CF98, CF99, CF100, CF101, CF102, and CF103 were adjusted to 20 µg-protein/ml by 50 mM Na_2_HPO_4_-NaH_2_PO_4_ buffer (pH 7.0), respectively, and those of CF104 and CF105 were adjusted to the value of 6.8 and 3.6 µg-protein/ml by adding 50 mM Na_2_HPO_4_-NaH_2_PO_4_ buffer (pH 7.0), respectively. Emission at 525 nm.

In the excitation spectral analysis measuring fluorescence emitted at 525 nm, all but CF104 (with a prominent peak at 413 nm) showed a prominent fluorescence peak at approximately 504 nm of excitation wavelength with a smaller peak at approximately 413 nm (Figure 2). To confirm the citrate concentration-dependence of the fluorescence signal, the excitation spectra of the nine chimeric proteins were measured in a buffer containing 0.05, 0.5, 5, 50, and 100 mM citrate. The fluorescence intensities that were excited at 504 nm (FI504) and emitted at 525 nm by CF97, CF99, CF100, CF102, CF103, CF104, and CF105 decreased upon citrate addition. Among these seven chimeric proteins, CF99 showed the highest fluorescence intensity. In contrast, FI504 by CF98 and CF101 increased upon citrate addition: the former showed higher fluorescence intensity. On the other hand, no such fluorescence signal changes were caused by the reaction of any of the cpFPs with 200 mM NaCl. In parallel experiments, to examine whether or not the smaller peak found at approximately 413 nm in the spectra was due to the fluorescence signal from cpFP, the excitation spectra of CF98 and CF99 were remeasured at 540 nm of emission wavelength instead of 525 nm. Despite such a change in emission wavelength, the peak at approximately 413 nm still appeared in the excitation spectra of CF98 and CF99 (data not shown). These results suggest that the small peak at 413 nm may not be attributed to other spectroscopic phenomena, e.g., Raman scattering, but to the fluorescence signal from CF98 and CF99.

We focused on CF98 as the cpFP-based citrate indicator for the analyses of the molecular and physicochemical properties because of the high fluorescence intensity, its clear citrate-concentration dependence, and its ideal detectable range for the intracellular citrate concentration.

### In Vitro characterization of CF98

**Figure 3:**
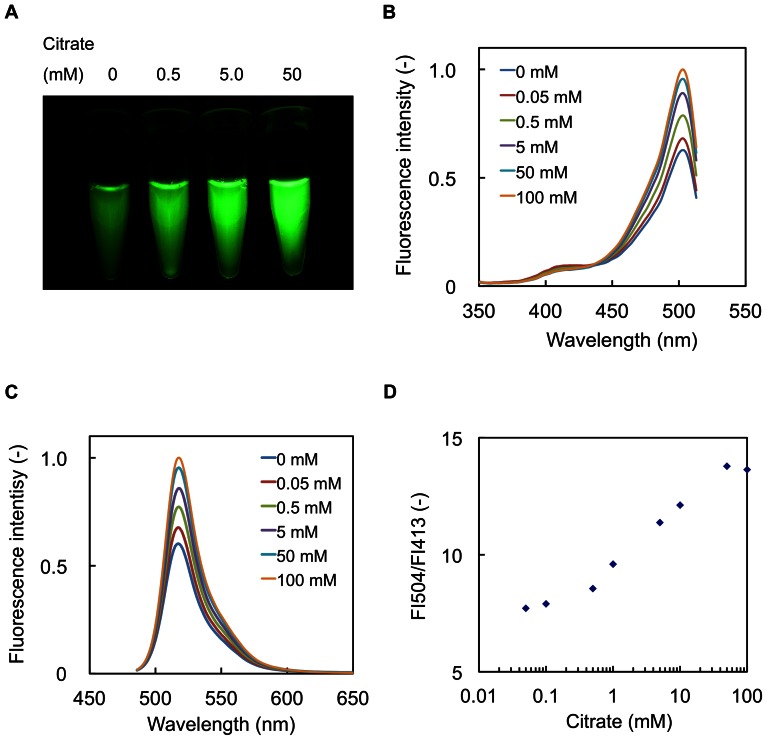
Fluorescence properties of CF98. A, Photoimages of CF98. Purified CF98 (3.0 mg-protein/ml) in 50 mM Na_2_HPO_4_-NaH_2_PO_4_ buffer (pH 7.0) containing the indicated citrate concentration was poured into 0.6-ml microtubes. The microtubes were then illuminated with 470 nm LED light for excitation, and photos were taken with a digital camera using a long-pass filter of 515 nm to exclude 470 nm LED light in a darkroom with constant exposure. B, Excitation spectra of CF98. Excitation spectra (emission at 525 nm) of CF98 were measured in 50 mM Na_2_HPO_4_-NaH_2_PO_4_ buffer (pH 7.0) containing the indicated citrate concentration. C, Emission spectra of CF98. Emission spectra (excitation at 475 nm) of CF98 were measured in 50 mM Na_2_HPO_4_-NaH_2_PO_4_ buffer (pH 7.0) containing the indicated citrate concentration. D, Fluorescence intensity ratio of CF98. The ratio of the fluorescence intensity at 504 nm (FI504) to that at 413 nm (FI413) was plotted against citrate concentration. Data points are averages of three independent experiments. Error bars indicate standard deviations.

The fluorescence changes of CF98 were rapidly observed (<1 msec) upon the addition of citrate in the range of 0.1 to 50 mM. The dependence of CF98 excitation and fluorescence emission was further tested by mixing of purified CF98 (3.0 mg protein/ml) with different concentrations of citrate (Figure 3A). Increase in the fluorescence intensities of CF98 was shown to be apparently proportional to the increasing concentrations of citrate in the range of 0.1 to 50 mM.

The excitation spectra of CF98 (emission at 525 nm) showed a prominent peak at 504 nm with a minor one at 413 nm (Figure 3B) and the emission spectra of CF98 (excitation at 475 nm) showed a peak at 518 nm (Figure 3C). As shown in Figure 3B, the major 504 nm peak was markedly enhanced, while the minor 413 nm peak was almost disappeared, as citrate concentrations increased, implying that the increase of citrate concentration leads to a ratiometric change in the excitation spectrum of CF98. The FI ratios (FI504/FI413) of CF98 were increased by the addition of increasing amounts of citrate (Figure 3D), but they were independent of protein concentrations of CF98 (data not shown). Thus, measuring the ratio of the fluorescence intensities of the peaks between at 413 nm and at 504 nm allows us to estimate the citrate concentrations independently of those of the fluorescent protein.

To examine the selectivity of CF98 toward citrate, we tested the effects of several organic acids on the excitation spectra of CF98 (Table 1). The fluorescence intensities of CF98 changed only upon the addition of 5 mM citrate and none of the other carboxylates (5 mM each) tested were able to induce any significant changes in the FI504/FI413 ratio of the fluorescence emitted at 525 nm. Table 1 also clearly indicates that CF98 can detect citrate even in the buffer containing other organic acids, such as isocitrate and malate. These findings strongly suggest that CF98 may be valuable for highly selective assay of citrate. The fluorescence intensities (excitation at 504 nm and emission at 525 nm) of CF98 were found to be sensitive to pH and metal ions (Figure S1 and Table S2, respectively) by measuring the excitation spectra of CF98 using a series of buffers (pH 6.5 to pH 8.0) containing each of various metal ions. These data will be of use in the formulation of the conditions of citrate assay with this specific fluorescent protein.

**Table 1:** Citrate-selective fluorescent property of CF98.

Carboxylate added	FI/504/FI413 of CF98	
	Citrate 0 mM	5 mM
None	7.13±0.195	12.0±0.523
Isocitrate	7.55±0.416	12.2±0.597
α-Ketoglutarate	6.90±0.175	12.2±0.364
Succinate	7.19±0.104	12.1±0.315
Fumarate	7.02±0.248	12.3±0.701
Malate	6.96±0.123	12.5±0.771
Oxaloacetate	6.69±0.309	12.0±0.477
Pyruvate	7.22±0.383	12.0±0.521

Fluorescence intensities (FIs) by purified recombinant CF98 protein were measured at 525 nm when excited at 504 nm (FI504) and 413 nm (FI413) in 50 mM Na_2_HPO_4_-NaH_2_PO_4_ buffer (pH 7.0) containing the indicated carboxylates (5 mM). The values are averages and standard deviations of three independent experiments.

### In vivo assay of citrate concentrations in E. coli

Using *E. coli* cells expressing the gene encoding the citrate carrier protein CitT [30], we tested the capability of CF98 to detect the *in vivo* changes of citrate concentrations. Since CitT of *E. coli* is a transporter exchanging extracellular citrate and intracellular carboxylate, it is presumed that the change in the extracellular citrate concentration would rapidly cause changes in the intracellular citrate concentration of *E. coli* cells expressing the gene encoding CitT.

Expression vectors for CF98 and CitT were constructed with pET-21d(+) and pRSFDuet-1, named pECF98 and pRCITT, respectively, and then used for co-transformation of *E.coli* BL21(DE3) to obtain *E. coli* BL21(DE3)/pECF98+pRCITT. *E. coli* BL21(DE3)/pECF98+pRSFDuet-1, BL21(DE3) carrying pRSFDuet-1 instead of the pRCITT plasmids, was used as a negative control in the *in vivo* fluorometric analysis. Aliquots of the cell suspension of *E. coli* BL21(DE3)/pECF98+pRCITT or *E. coli* BL21(DE3)/pECF98+pRSFDuet-1 were poured into a cuvette and placed in a fluorescence spectrometer to start measurement. Buffers containing different citrate concentrations (0 to 2.5 mM) were added into aliquots of the cell suspension after 50 s and the changes in fluorescence intensity were sequentially determined.

**Figure 4:**
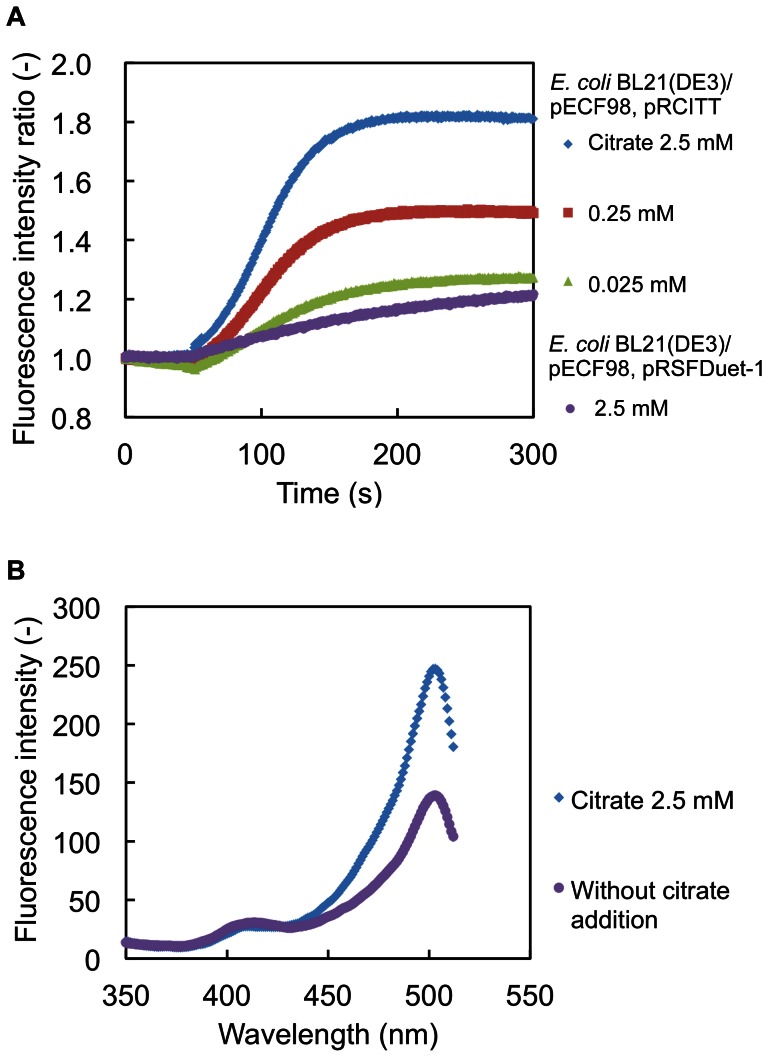
*In vivo* citrate detection using CF98 in *E.* *coli* expressing the gene encoding citrate carrier CitT. A, Time courses of the fluorescence intensities of CF98 from *E. coli* BL21(DE3)/pECF98+pRCITT and *E. coli* BL21(DE3)/pECF98+pRSFDuet-1. The fluorescence intensities of CF98 from aliquots of the cell suspension were measured in 50 mM Na_2_HPO_4_-NaH_2_PO_4_ buffer (pH 7.0). At 50 s after starting the measurements, the citrate-containing buffer was added to change the extracellular citrate concentration to the indicated citrate concentrations in the cuvette. The fluorescence intensities (excitation at 504 nm, emission at 525 nm) at each time point in three independent experiments were averaged. B, Excitation spectrum (emission at 525 nm) of CF98 from aliquots of the cell suspension of *E. coli* BL21(DE3)/pECF98+pRCITT was measured 150 s after the addition of the citrate-containing buffer to change the citrate concentration from 0 to 2.5 mM. The control excitation spectrum was measured without addition of citrate.

The time courses of the changes in the fluorescence intensities of CF98 (excitation at 504 nm and emission at 525 nm) from aliquots of the cell suspension of *E. coli* BL21(DE3)/pECF98+pRCITT and *E. coli* BL21(DE3)/pECF98+pRSFDuet-1 are shown in Figure 4A. The fluorescence intensities of CF98 from aliquots of the cell suspension of *E. coli* BL21(DE3)/pECF98+pRCITT increased with the incremental changes of concentrations of citrate extracellularly added. The fluorescence intensities of CF98 from aliquots of the cell suspension of *E. coli* BL21(DE3)/pECF98+pRCITT were maximized at 150 s after the addition of the citrate-containing buffer. The increase in fluorescence intensities was also confirmed in the excitation spectra (Figure 4B). In the excitation spectra of CF98 from aliquots of the cell suspension of *E. coli* BL21(DE3)/pECF98+pRCITT, two peaks at 413 and 504 nm were detected, the minor 413 nm peak diminished, and the major 504 nm peak was enhanced with the citrate addition, analogously to the *in vitro* analysis with purified CF98, as shown in Figure 3B. In contrast to these results, no significant change in the fluorescence intensities of CF98 with aliquots of the cell suspension of *E. coli* BL21(DE3)/pECF98+pRSFDuet-1 was observed even though the extracellular citrate concentrations changed with the addition of the citrate-containing buffer.

### Intracellular pH-dependent change of CF98 fluorescence intensities and its correction

Since CF98 fluorescence is sensitive *in vitro* to changes in pH (Figure S1), it is important for accurate quantitation of citrate to isolate the fluorescence changes induced by the changes in the citrate concentrations from those induced by pH changes. The intracellular pH of *E. coli* was determined using the fluorescent pH indicator dye, SNARF-5F 5-(and-6)-carboxylic acid, acetoxymethyl ester, acetate (SNARF-5F-AM ester; Molecular Probes, OR, USA), in accordance with the manufacturer's protocol. The SNARF-5F signals were calibrated by the high-K^+^/nigericin method [31].

The excitation spectra (emission at 630 nm) of the aliquots of cell suspension of SNARF-5F-loaded *E. coli* BL21(DE3)/pECF98+pRCITT diluted with each pH calibration solution showed marked changes in the fluorescence signals of SNARF-5F due to pH changes (Figure S2). Since the emission peak of CF98 was found at 518 nm (Figure 3C) and its fluorescence signals hardly overlapped with those of SNARF-5F, the presence of the former in the *E. coli* cells is negligible for determination of the fluorescence emitted from SNARF-5F. The fluorescence spectra of the SNARF-5F-loaded *E. coli* cells showed one excitation peak at 577 nm. A calibration curve for the intracellular pH of *E. coli* was constructed by plotting the values of the fluorescence intensities excited at 577 nm and emitted at 630 nm against the values of pH (Figure S3).

The excitation spectrum of SNARF-5F from aliquots of the cell suspension of SNARF-5F-loaded *E. coli* BL21(DE3)/pECF98+pRCITT diluted with 50 mM Na_2_HPO_4_-NaH_2_PO_4_ buffer (pH 7.0) and that of the *E. coli* cell suspension at 150 s after the addition of the citrate-containing buffer to give a final concentration of 2.5 mM citrate are also shown in Figure S2. The values of pH inside the *E. coli* cells before and after the citrate addition were barely different (pH 6.59±0.0608 and 6.56±0.0516, respectively) (Figure S2) based on the calculation with the calibration curve (Figure S3).

**Figure 5:**
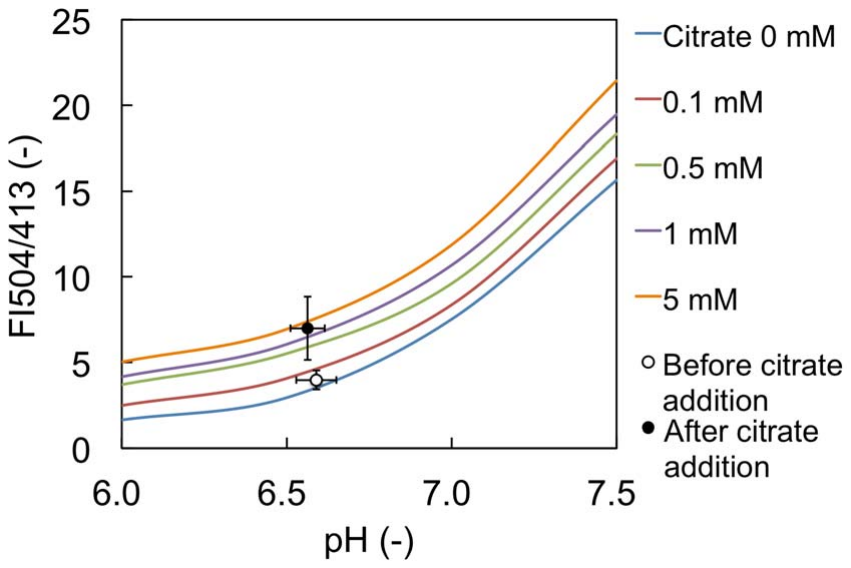
Correction of fluorescence intensities for intracellular pH changes in *E.* *coli* using SNARF-5F. The ratios of the fluorescence intensities of CF98 (FI504/FI413) were measured using purified CF98 *in vitro* and are represented by solid lines. The pH-corrected FI504/FI413 values of CF98 from aliquots of the cell suspension of *E. coli* BL21(DE3)/pECF98+pRCITT before and after citrate addition are also plotted and represented by open and closed circles, respectively. Data points are averages of three independent experiments. Error bars indicate standard deviations.

The FI504/FI413 ratios of the fluorescence intensities of CF98 allow us to determine the citrate concentrations as shown in Figure 3D. Plotting the FI504/FI413 ratios measured *in vitro* with purified CF98 against pH enabled us to correct the fluorescence signals of CF98 for pH (Figure 5). Furthermore, in Figure 5, the FI504/FI413 values of CF98 in the *E. coli* BL21(DE3)/pECF98+pRCITT cells before and after citrate addition were calculated from the results shown in Figure 4B and plotted against each of the corresponding intracellular pH values calculated in Figure S1. The pH-corrected FI504/FI413 values of CF98 in the *E. coli* cells showed a clear increase in response to the elevation of intracellular citrate concentrations, indicating that the newly developed citrate detection system is useful for *in vivo* as well as *in vitro* quantitative assay of citrate.

## Discussion

In this study, we developed the fluorescent-protein-based indicators for citrate by inserting cpFP in the flexible region of CitAP, the periplasmic domain of a sensor histidine kinase protein CitA that can bind to citrate with high specificity, and examined in detail the molecular and physicochemical properties of CF98 as the most promising citrate indicator. The fluorescent signals of CF98 appeared to be ratiometric: upon citrate addition, the excitation peak at 504 nm increased proportionally to the decrease in the excitaion peak at 413 nm. The ratiometric properties that also have been reported for ratiometric Pericam [19], Hyper [20], and Perceval [22] are essential for intracellular indicators because they permit built-in normalization of the fluorescence signals irrespective of variations in the concentrations of the indicator proteins. CF98 can detect citrate in the range of 0.1 to 50 mM (Figure 3D) with high selectivity. This detectable range is ideal for the determination of the intracellular citrate concentration in microorganisms, according to the reported data on metabolome analyses [32], [33], [34]. For example, Bennett et al. reported that the intracellular citrate concentration of exponentially growing *E. coli* cells in glucose-fed batch culture is 1.96 mM, that of growing with acetate is 2.32 mM, and that of growing with acetate is 21.9 mM [33], and that of *Saccharomyces cerevisiae* cells is determined to be 5.2 mM [34].

We demonstrated the successful application of CF98 to the detection of *in vivo* changes in citrate concentration by using *E. coli* cells expressing the gene encoding a citrate carrier CitT. Since CitT functions as a transporter exchanging extracellular citrate and succinate [30], changing the extracellular citrate concentration was predicted to cause changes in the intracellular citrate concentration, in turn leading to the increase in the fluorescence intensities of CF98 in *E. coli* cells co-expressing the genes encoding CF98 and CitT. As shown in Figure 4, as expected, the fluorescence intensities of CF98 in *E. coli* BL21(DE3)/pECF98+pRCITT were remarkably increased by the addition of the citrate-containing buffer to the cell suspension, in contrast to no significant change in *E. coli* BL21(DE3)/pECF98+pRSFDuet-1.

The fluorescence signals of CF98 were found to be sensitive to pH changes and the existence of some metal ions such as Fe^2+^ and Ni^2+^. Similarly, for all other cpFP-based indicators ever developed, sensitivities of their fluorescence signals to intracellular pH have imposed problematic technical limitations on their practical uses [19], [20], [22]. Many metabolic perturbations also affect intracellular pH and thus these variation factors must be taken into account for the applications of CF98 to *in vitro* and *in vivo* of their ligands. We measured the changes in intracellular pH using the fluorescent pH indicator dye SNARF-5F and corrected for the pH effects on CF98 signals by measuring the ratiometric plots of the pH-dependent CF98 fluorescence signals. As a result, the changes in the intracellular citrate concentrations after the addition of citrate-containing buffer to the cell suspension was clearly shown (Figure 5), strongly suggesting that CF98 is an eligible indicator for *in vivo* as well as *in vitro* quantitative detection of citrate.

In addition to its wide array of industrial and medical uses, citrate is an important intermediate in TCA cycle and also an essential regulatory molecule in glycolysis and fatty acid synthesis [4], [35]. Our newly developed cpFP-based citrate indicator can be effectively employed to follow the metabolic activities of cells in question to understand dynamic intracellular events in which citrate is involved in living cells and to explore the mechanism of citrate production.

## Materials and Methods

### Strains and cultivation conditions

*E. coli* strains JM109 and BL21(DE3) were used as the hosts for the plasmid construction and protein expression, respectively. *K. pneumoniae* NBRC 13541 was used as the source of the gene encoding the sensor histidine kinase CitA. The gene encoding the citrate carrier (CitT: gene ID, 949070) was obtained from the genomic DNA of *E. coli* W3110 [30]. *E. coli* and *K. pneumoniae* strains were grown routinely at 37°C in LB medium with appropriate antibiotics.

### Gene expression and protein purification

Recombinant proteins were produced using *E. coli* BL21(DE3) as the host and the pET vector system (Novagen), and purified by Ni^2+^-affinity chromatography using HisTrap HT columns (GE Healthcare). The cell-free extracts were loaded onto HisTrap HT columns that have been equilibrated with TNI5 buffer (20 mM Tris-HCl, pH 7.9, 500 mM NaCl, and 5 mM imidazole). Weakly binding proteins were removed by washing with five bed volumes of TNI5 buffer. Elution was performed with TNI500 buffer containing 500 mM imidazole and the buffer was subsequently exchanged to 50 mM Na_2_HPO_4_-NaH_2_PO_4_ buffer (pH 7.0) by gel filtration with Sephadex G-25 (PD-10 column, GE Healthcare). Protein concentrations were determined by the Bradford method using Coomassie Protein Assay Reagent (Thermo Scientific, Illinois, USA) with bovine serum albumin as the standard.

### Construction of CitAP- and cpFP-expression plasmids

To generate fluorescent-protein-based citrate indicators, the DNA sequence coding for the periplasmic domain of sensor histidine kinase CitA (CitAP) (45–176 residues of CitA) was amplified from the total DNA of *K. pneumoniae* NBRC 13541 by PCR with the specific primers Pr1 and Pr2 shown in Table S1. The amplified DNA fragment was digested with *Nco* I and *Xho* I, and the resulting restriction fragment was inserted into *Nco* I/*Xho* I sites of pET-21d(+) vector for construction of the expression plasmid pECitAP.

To generate the cpFPs, two fragments of the enhanced green fluorescent protein (EGFP) coding sequence were amplified from the plasmid pBEGFP-F [36], [37] by PCR with the specific primers shown in Table S1: Pr3 and Pr4 for the fragment of residues 145–238 of EGFP; Pr5 and Pr6 for the fragment of residues 2–144 of EGFP (amino acid residue numbers correspond to those of the standard GFP). The primers Pr3 and Pr6 have short DNA sequences coding for amino acid linkers, -SAG- and -GT-, respectively, located between cpFP and CitAP, and also have restriction sites, *Pst* I and *Kpn* I, respectively. The primers Pr4 and Pr5 have a sequence coding for a linker (-VDGGSGGTG- between the original C and N termini), allowing complementary fragment annealing in the following elongation reactions. These linker sequences (-SAG-, -GT-, and -VDGGSGGTG-) have been optimized for the ratiometric calcium indicator, Pericam [19]. The two fragments amplified with Pr3/Pr4 and Pr5/Pr6 were mixed, annealed, and elongated by PCR with Pr3 and Pr6. The resulting fragment encoding a circularly permuted cpFP was amplified by PCR with Pr3 and Pr6, digested with *Pst* I and *Kpn* I, and the restriction fragment covering the coding region for cpFP was inserted into pUC19 (Takara Bio). Since the mutation (F46L) was essential for accelerating the chromophore maturation in accordance with the report of the fluorescent protein Venus [38] and the mutations (T65G, V68L, S72A, H148D, T203F) were optimized for the ratiometric Pericam [19], several mutations (F46L, T65G, V68L, S72A, H148D, T203F) were introduced into the cpFP coding sequence by site-directed mutagenesis with primers Pr7-12 (Table S1).

### Generation of fluorescent-protein-based indicators for citrate

To generate fluorescent-protein-based indicators for citrate, the cpFP coding sequence was inserted into the flexible region (between any of the residues 97–105) of the CitAP coding sequence. The chimeric proteins to be obtained have the following structures: CitA residues 45-*N*-SAG-cpFP-GT-CitAP residues (*N*+1)-176-His_6_, where *N* is the location number of any one of the CitA residues between 97 and 105, and SAG and GT are short amino acid linkers between CitAP and cpFP. To amplify the fragment of the SAG-cpFP-GT coding sequence, the primers Pr13 and Pr14 were used. To amplify the fragment of the CitAP coding sequence, Pr1, Pr2, and Pr15-Pr32 were used. PCR fragments of CitAP residues 45-*N*, SAG-cpFP-GT, and CitAP residues (*N*+1)-176 were mixed, and amplified by PCR with Pr1 and Pr2. The fragments containing the region encoding the fluorescent-protein-based indicators for citrate were digested with *Nco* I and *Xho* I, and inserted into pET-21d(+) for the construction of the CitAP and cpFP expression plasmids (pECF97 to pECF105) that were used for transformation of *E. coli* BL21(DE3) to produce the chimeric proteins. The chimeric proteins were named CF97 to CF105 according to the location number *N*. The full nucleotide and protein sequences of CF98 are given in Figure S4.

### Fluorometric analysis

Chimeric proteins were purified as His-tagged proteins from the recombinant *E. coli* BL21(DE3) cells expressing each of the chimeric proteins. The fluorescence spectra of the purified chimeric proteins were measured in 50 mM Na_2_HPO_4_-NaH_2_PO_4_ buffer (pH 7.0) (control) supplemented with or without citrate using a fluorescence spectrometer (FP-6200, JASCO Corporation) that was equipped with a water thermostated cell holder with a stirrer (STR-312, JASCO Corporation) and a sample holder lid with a syringe port (CSP-622, JASCO Corporation). Excitation spectra were measured at 25°C by the spectrometer under the following conditions: emission wavelength, 525 nm; wavelength range, 350–512 nm; spectral bandwidth, 5 nm; response, medium; wavelength scan speed, 2000 nm/min; gain, medium. Emission spectra were measured under the following conditions: excitation wavelength, 475 nm; wavelength range, 482–650 nm; spectral bandwidth, 5 nm; response, medium; wavelength scan speed, 2000 nm/min; gain, medium.

### In vivo assay of citrate concentration in E. coli

To express the gene encoding CF98 in the *in vivo* assay of the changes in the intracellular citrate concentration, *E. coli* BL21(DE3) cells carrying pECF98 and pRCITT or pRSFDuet-1 (Novagen) as the negative control were used. To express the genes encoding CF98 and CitT, *E. coli* BL21(DE3)/pECF98+pRCITT cells were cultivated and the culture of *E. coli* BL21(DE3)/pECF98+pRSFDuet-1 was used as the control. After the induction of the genes, *E. coli* cells were harvested by centrifugation, washed twice with 50 mM Na_2_HPO_4_-NaH_2_PO_4_ buffer (pH 7.0), and resuspended in the same buffer. Aliquots of the *E. coli* cell suspension were used for the detection of the changes in the intracellular citrate concentration and the intracellular pH calibration.

The plasmid pRCITT, an expression vector of the gene encoding citrate carrier (CitT; Gene ID, 949070) in *E. coli* K-12 [30], was constructed as follows. A fragment of the gene encoding CitT was amplified from the total DNA extracted from *E. coli* W3110 by PCR with the specific primers Pr33 and Pr34, shown in Table S1, and the amplified fragment was digested with *Nde* I and *Mfe* I. The resulting restriction fragment was inserted into the *Nde* I/*Mfe* I sites of pRSFDuet-1 (Novagen) for the construction of the plasmid pRCITT. Aliquots of the cell suspension of *E. coli* BL21(DE3)/pECF98+pRCITT and *E. coli* BL21(DE3)/pECF98+pRSFDuet-1 (negative control) were applied to the fluorometric analysis. To measure the changes in the fluorescence intensities of CF98 due to the changes in the intracellular citrate concentration, 100 µl of the cell suspension and 1,875 µl of 50 mM Na_2_HPO_4_-NaH_2_PO_4_ buffer (pH 7.0) were poured into a cuvette and placed in the spectrometer. During the measurement of the fluorescence intensities, 25 µl of 200 mM citrate-containing 50 mM Na_2_HPO_4_-NaH_2_PO_4_ buffer (pH 7.0) were added to the aliquots of the cell suspension.

### Intracellular pH calibration

To confirm whether or not the intracellular pH changes with the addition of the citrate-containing buffer to the *E. coli* cell suspension, the intracellular pH of *E. coli* was determined using the fluorescent pH indicator dye SNARF-5F 5-(and-6)-carboxylic acid, acetoxymethyl ester, acetate (SNARF-5F-AM ester; Molecular Probes, OR, USA), basically in accordance with the manufacturer's protocol.

The solution of SNARF-5F-AM ester diluted with dimethyl sulfoxide (DMSO) was added to one milliliter of the *E. coli* cell suspension to give a final concentration of 10 µM. The mixture was then incubated at room temperature for 3 h with shading. After incubation with the SNARF-5F-AM, *E. coli* cells were washed twice and resuspended with 50 mM Na_2_HPO_4_-NaH_2_PO_4_ buffer (pH 7.0).

The SNARF-5F signals were calibrated by the high-K^+^/nigericin method [31]. The ionophore nigericin (Molecular Probes) is generally used for intracellular pH calibration in the presence of 100–150 mM K^+^ to equilibrate the intracellular pH with the controlled extracellular pH. As calibration solutions, a series of 50 mM Na_2_HPO_4_-NaH_2_PO_4_ buffers containing 10 µg/ml nigericin and 150 mM KCl with pHs ranging from 6.0 to 8.0 were prepared. For intracellular pH calibration, 50 µl of the suspension of SNARF-5F-loaded *E. coli* cells and 950 µl of 50 mM Na_2_HPO_4_-NaH_2_PO_4_ buffers containing 10 µg/ml nigericin and 150 mM KCl were mixed and incubated at 25°C for 10 min. After the incubation, the fluorescence spectra (emission at 630 nm) of SNARF-5F loaded in the cells were measured using a fluorescence spectrometer.

## Supporting Information

Table S1 **Primers used for generation of cpFP-based indicators for citrate.**
(DOCX)

Table S2 **Metal ion sensitivity of CF98.**
(DOCX)

Figure S1 **pH sensitivity of CF98.** The fluorescence intensities (excitation at 504 nm and emission at 525 nm) of CF98 were measured using a series of buffers prepared with pHs ranging from 6.5 to 8.0 containing the indicated citrate concentrations. CF98 is sensitive to pH, but the magnitude relationship between fluorescence intensity and citrate concentration is maintained.
(TIF)

Figure S2 **Effects of pH on the excitation spectra from aliquots of the cell suspension of SNARF-5F-loaded** ***E. coli*** **BL21(DE3)/pECF98+pRCITT.** The solid lines represent the excitation spectra of SNARF-5F from the cell suspensions diluted with calibration solutions for each of the indicated values of pH. The dotted lines represent the excitation spectra of SNARF-5F from the cell suspensions measured at 50 s after addition of the citrate-containing buffer to give the final concentration of 2.5 mM. Emission at 630 nm.
(TIF)

Figure S3 **Calibration curve of intracellular pH versus fluorescence intensity of SNARF-5F-loaded** ***E. coli*** **cells.** The pH calibration curve constructed by plotting fluorescence intensities (excitation at 577 nm and emission at 630 nm) of SNARF-5F from aliquots of the cell suspension of SNARF-5F-loaded *E. coli* BL21(DE3)/pECF98+pRCITT diluted with each pH calibration solution against pH.
(TIF)

Figure S4 **The full nucleotide and protein sequences of CF98.**
(TIFF)

## References

[1] Krebs HA (1940) The citric acid cycle: A reply to the criticisms of F. L. Breusch and of J. Thomas. Biochem J 34: 460–463. 16747181 10.1042/bj0340460 PMC1265298

[2] Kirimura K, Honda Y, Hattori T (2011) Citric acid. In: Moo-Young M, editor. Comprehensive Biotechnology. Second Edition: Elsevier. pp. 135–142.

[3] Demain AL, Sanchez S (2011) Microbial synthesis of primary metabolites: current trends and future prospects. In: El-Mansi EMT, Bryce CFA, Dahhou B, Sanchez S, Demain AL, et al.., editors. Fermentation Microbiology and Biotechnology. Third Edition: CRC Press. pp. 77–100.

[4] Geelen MJ, Schmitz MG (1993) The role of citrate in the regulation of hepatic fatty acid synthesis by insulin and glucagon. Horm Metab Res 25: 525–527. 7903266 10.1055/s-2007-1002166

[5] Tosukhowong P, Borvonpadungkitti S, Prasongwatana V, Tungsanga K, Jutuporn S, et al (2002) Urinary citrate excretion in patients with renal stone: roles of leucocyte ATP citrate lyase activity and potassium salts therapy. Clin Chim Acta 325: 71–78. 12367768 10.1016/s0009-8981(02)00254-1

[6] Fricke ST, Rodriguez O, Vanmeter J, Dettin LE, Casimiro M, et al (2006) *In vivo* magnetic resonance volumetric and spectroscopic analysis of mouse prostate cancer models. Prostate 66: 708–717. 16425198 10.1002/pros.20392

[7] Newman RH, Fosbrink MD, Zhang J (2011) Genetically encodable fluorescent biosensors for tracking signaling dynamics in living cells. Chem Rev 111: 3614–3666. 21456512 10.1021/cr100002u PMC3092831

[8] Kirimura K, Ogawa S, Hattori T, Kino K (2006) Expression analysis of alternative oxidase gene (*aox1*) with enhanced green fluorescent protein as marker in citric acid-producing *Aspergillus niger* . J Biosci Bioeng 102: 210–214. 17046535 10.1263/jbb.102.210

[9] Hattori T, Honda Y, Kino K, Kirimura K (2008) Expression of alternative oxidase gene (*aox1*) at the stage of single-cell conidium in citric acid-producing *Aspergillus niger* . J Biosci Bioeng 105: 55–57. 18295720 10.1263/jbb.105.55

[10] Honda Y, Kobayashi K, Kirimura K (2011) Increases in gene-targeting frequencies due to disruption of *kueA* as a *ku80* homolog in citric acid-producing *Aspergillus niger* . Biosci Biotechnol Biochem 75: 1594–1596. 21821927 10.1271/bbb.110015

[11] Bermejo C, Ewald JC, Lanquar V, Jones AM, Frommer WB (2011) *In VIVO* biochemistry: quantifying ion and metabolite levels in individual cells or cultures of yeast. Biochem J 438: 1–10. 21793803 10.1042/BJ20110428

[12] Evellin S, Mongillo M, Terrin A, Lissandron V, Zaccolo M (2004) Measuring dynamic changes in cAMP using fluorescence resonance energy transfer. Methods Mol Biol 284: 259–270. 15173622 10.1385/1-59259-816-1:259

[13] Imamura H, Nhat KP, Togawa H, Saito K, Iino R, et al (2009) Visualization of ATP levels inside single living cells with fluorescence resonance energy transfer-based genetically encoded indicators. Proc Natl Acad Sci USA 106: 15651–15656. 19720993 10.1073/pnas.0904764106 PMC2735558

[14] Dulla C, Tani H, Okumoto S, Frommer WB, Reimer RJ, et al (2008) Imaging of glutamate in brain slices using FRET sensors. J Neurosci Methods 168: 306–319. 18160134 10.1016/j.jneumeth.2007.10.017 PMC2267481

[15] Fehr M, Frommer WB, Lalonde S (2002) Visualization of maltose uptake in living yeast cells by fluorescent nanosensors. Proc Natl Acad Sci USA 99: 9846–9851. 12097642 10.1073/pnas.142089199 PMC125039

[16] Fehr M, Lalonde S, Ehrhardt DW, Frommer WB (2004) Live imaging of glucose homeostasis in nuclei of COS-7 cells. J Fluoresc 14: 603–609. 15617267 10.1023/b:jofl.0000039347.94943.99

[17] Ewald JC, Reich S, Baumann S, Frommer WB, Zamboni N (2011) Engineering genetically encoded nanosensors for real-time *in vivo* measurements of citrate concentrations. PLoS One 6: e28245. 22164251 10.1371/journal.pone.0028245 PMC3229521

[18] Baird GS, Zacharias DA, Tsien RY (1999) Circular permutation and receptor insertion within green fluorescent proteins. Proc Natl Acad Sci USA 96: 11241–11246. 10500161 10.1073/pnas.96.20.11241 PMC18018

[19] Nagai T, Sawano A, Park ES, Miyawaki A (2001) Circularly permuted green fluorescent proteins engineered to sense Ca^2+^ . Proc Natl Acad Sci USA 98: 3197–3202. 11248055 10.1073/pnas.051636098 PMC30630

[20] Belousov VV, Fradkov AF, Lukyanov KA, Staroverov DB, Shakhbazov KS, et al (2006) Genetically encoded fluorescent indicator for intracellular hydrogen peroxide. Nat Methods 3: 281–286. 16554833 10.1038/nmeth866

[21] Markvicheva KN, Bilan DS, Mishina NM, Gorokhovatsky AY, Vinokurov LM, et al (2011) A genetically encoded sensor for H_2_O_2_ with expanded dynamic range. Bioorg Med Chem 19: 1079–1084. 20692175 10.1016/j.bmc.2010.07.014

[22] Berg J, Hung YP, Yellen G (2009) A genetically encoded fluorescent reporter of ATP:ADP ratio. Nat Methods 6: 161–166. 19122669 10.1038/nmeth.1288 PMC2633436

[23] Bott M, Meyer M, Dimroth P (1995) Regulation of anaerobic citrate metabolism in *Klebsiella pneumoniae* . Mol Microbiol 18: 533–546. 8748036 10.1111/j.1365-2958.1995.mmi_18030533.x

[24] Bott M (1997) Anaerobic citrate metabolism and its regulation in enterobacteria. Arch Microbiol 167: 78–88. 9133329

[25] Kaspar S, Perozzo R, Reinelt S, Meyer M, Pfister K, et al (1999) The periplasmic domain of the histidine autokinase CitA functions as a highly specific citrate receptor. Mol Microbiol 33: 858–872. 10447894 10.1046/j.1365-2958.1999.01536.x

[26] Gerharz T, Reinelt S, Kaspar S, Scapozza L, Bott M (2003) Identification of basic amino acid residues important for citrate binding by the periplasmic receptor domain of the sensor kinase CitA. Biochem 42: 5917–5924. 12741850 10.1021/bi0340595

[27] Reinelt S, Hofmann E, Gerharz T, Bott M, Madden DR (2003) The structure of the periplasmic ligand-binding domain of the sensor kinase CitA reveals the first extracellular PAS domain. J Biol Chem 278: 39189–39196. 12867417 10.1074/jbc.M305864200

[28] Sevvana M, Vijayan V, Zweckstetter M, Reinelt S, Madden DR, et al (2008) A ligand-induced switch in the periplasmic domain of sensor histidine kinase CitA. J Mol Biol 377: 512–523. 18258261 10.1016/j.jmb.2008.01.024

[29] McNicholas S, Potterton E, Wilson KS, Noble ME (2011) Presenting your structures: the CCP4mg molecular-graphics software. Acta Crystallogr D Biol Crystallogr 67: 386–394. 21460457 10.1107/S0907444911007281 PMC3069754

[30] Pos KM, Dimroth P, Bott M (1998) The *Escherichia coli* citrate carrier CitT: a member of a novel eubacterial transporter family related to the 2-oxoglutarate/malate translocator from spinach chloroplasts. J Bacteriol 180: 4160–4165. 9696764 10.1128/jb.180.16.4160-4165.1998 PMC107412

[31] Thomas JA, Buchsbaum RN, Zimniak A, Racker E (1979) Intracellular pH measurements in Ehrlich ascites tumor cells utilizing spectroscopic probes generated in situ. Biochem 18: 2210–2218. 36128 10.1021/bi00578a012

[32] Albe KR, Butler MH, Wright BE (1990) Cellular concentrations of enzymes and their substrates. J Theor Biol 143: 163–195. 2200929 10.1016/s0022-5193(05)80266-8

[33] Bennett BD, Kimball EH, Gao M, Osterhout R, Van Dien SJ, et al (2009) Absolute metabolite concentrations and implied enzyme active site occupancy in *Escherichia coli* . Nat Chem Biol 5: 593–599. 19561621 10.1038/nchembio.186 PMC2754216

[34] Lagunas R, Gancedo C (1983) Role of phosphate in the regulation of the Pasteur effect in *Saccharomyces cerevisiae* . Eur J Biochem 137: 479–483. 6229402 10.1111/j.1432-1033.1983.tb07851.x

[35] Gnoni GV, Priore P, Geelen MJ, Siculella L (2009) The mitochondrial citrate carrier: metabolic role and regulation of its activity and expression. IUBMB Life 61: 987–994. 19787704 10.1002/iub.249

[36] Chiu W, Niwa Y, Zeng W, Hirano T, Kobayashi H, et al (1996) Engineered GFP as a vital reporter in plants. Curr Biol 6: 325–330. 8805250 10.1016/s0960-9822(02)00483-9

[37] Maruyama J, Nakajima H, Kitamoto K (2001) Visualization of nuclei in *Aspergillus oryza*e with EGFP and analysis of the number of nuclei in each conidium by FACS. Biosci Biotech Biochem 65: 1504–1510. 10.1271/bbb.65.1504 11515532

[38] Nagai T, Ibata K, Park ES, Kubota M, Mikoshiba K, et al (2002) A variant of yellow fluorescent protein with fast and efficient maturation for cell-biological applications. Nat Biotech 20: 87–90. 10.1038/nbt0102-87 11753368

