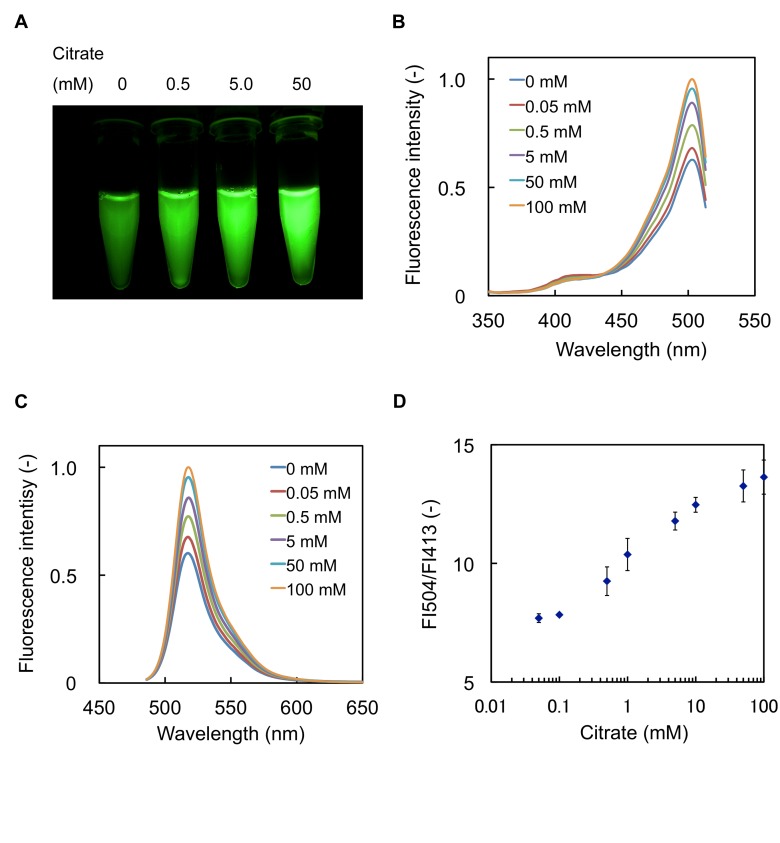# Correction: Generation of Circularly Permuted Fluorescent-Protein-Based Indicators for In Vitro and In Vivo Detection of Citrate

**DOI:** 10.1371/annotation/beae09ab-07c8-43de-b2de-a7e8368b77f6

**Published:** 2013-11-13

**Authors:** Yuki Honda, Kohtaro Kirimura

The standard error bars are missing from the graph of Figure 3D. Please see the corrected Figure 3 here: 

**Figure:**