# Supplementary material for: Generation of Circularly Permuted Fluorescent-Protein-Based Indicators for In Vitro and In Vivo Detection of Citrate
Source: PLoS One. 2013 May 22;8(5):e64597. doi: 10.1371/journal.pone.0064597 (PMC3661591; doi:10.1371/journal.pone.0064597)
Supplement: Table S1 — Primers used for generation of cpFP-based indicators for citrate. (DOCX) [file pone.0064597.s001.docx]

| **Table S1** **Primers used for generation of cpFP-based indicators for citrate** | | | |
| --- | --- | --- | --- |
| No. | Target | | Sequence (5’—3’) * |
| Pr1 | CitA (45-176) | | CCGCCATGGACATTACCGAGGAGCGTCTG *1 |
| Pr2 | CitA (45-176) | | TTACTCGAGTTGCTCAATGGTATAGCC *2 |
| Pr3 | EGFP (145-238) H148D | | AAATCTGCAGGCTACAACAGCGATAACGTC *3 |
| Pr4 | EGFP (145-238) | | GGTGCCACCGCTACCGCCGTCCACCTTGTACAGCTCGTC |
| Pr5 | EGFP (2-144) | | GGCGGTAGCGGTGGCACCGGCAGCAAGGGCGAGGAG |
| Pr6 | EGFP (2-144) | | AAAGGTACCGTTGTACTCCAGCTTGTG *4 |
| Pr7 | EGFP F46L | | GACCCTGAAGCTGATCTGCACCA |
| Pr8 | EGFP F46L | | TGGTGCAGATCAGCTTCAGGGTC |
| Pr9 | EGFP T203F | | CTACCTGAGCTTCCAGTCCGCCC |
| Pr10 | EGFP T203F | | GGGCGGACTGGAAGCTCAGGTAG |
| Pr11 | EGFP T65G V68L S72A | | TACGGCCTGCAGTGCTTCGCCCGCTAC |
| Pr12 | EGFP T65G V68L S72A | | AAGCACTGCAGGCCGTAGCCTAACGTG |
| Pr13 | cpFP | | TCTGCAGGCTACAACAGCG |
| Pr14 | cpFP | | GGTACCGTTGTACTCCAGCTTG |
| Pr15 | CF97 | CitAP (-97) | GCTGTTGTAGCCTGCAGACACTGACACGTAGCTTTTAG |
| Pr16 | CF97 | CitAP (98-) | TGGAGTACAACGGTACCCGCAAAGGCTCGCTGGGATCG |
| Pr17 | CF98 | CitAP (-98) | GCTGTTGTAGCCTGCAGAGCGCACTGACACGTAGCTT |
| Pr18 | CF98 | CitAP (99-) | TGGAGTACAACGGTACCAAAGGCTCGCTGGGATCGTC |
| Pr19 | CF99 | CitAP (-99) | GCTGTTGTAGCCTGCAGATTTGCGCACTGACACGTAGC |
| Pr20 | CF99 | CitAP (100-) | TGGAGTACAACGGTACCGGCTCGCTGGGATCGTCGC |
| Pr21 | CF100 | CitAP (-100) | GCTGTTGTAGCCTGCAGAGCCTTTGCGCACTGACACG |
| Pr22 | CF100 | CitAP (101-) | TGGAGTACAACGGTACCTCGCTGGGATCGTCGCTG |
| Pr23 | CF101 | CitAP (-101) | GCTGTTGTAGCCTGCAGACGAGCCTTTGCGCACTGAC |
| Pr24 | CF101 | CitAP (102-) | TGGAGTACAACGGTACCCTGGGATCGTCGCTGCGCGG |
| Pr25 | CF102 | CitAP (-102) | CTGTTGTAGCCTGCAGACAGCGAGCCTTTGCGCAC |
| Pr26 | CF102 | CitAP (103-) | TGGAGTACAACGGTACCGGATCGAGCCTGCGTGGTAA |
| Pr27 | CF103 | CitAP (-103) | CTGTTGTAGCCTGCAGATCCCAGCGAGCCTTTGCGCAC |
| Pr28 | CF103 | CitAP (104-) | TGGAGTACAACGGTACCAGCTCGCTGCGTGGTAAATCG |
| Pr29 | CF104 | CitAP (-104) | CTGTTGTAGCCTGCAGACGATCCCAGCGAGCCTTTG |
| Pr30 | CF104 | CitAP (105-) | TGGAGTACAACGGTACCTCGCTGCGTGGTAAATCGCC |
| Pr31 | CF105 | CitAP (-105) | CTGTTGTAGCCTGCAGACGACGATCCCAGCGAGCCTT |
| Pr32 | CF105 | CitAP (106-) | TGGAGTACAACGGTACCCTGCGCGGTAAATCGCC |
| Pr33 | CitT |  | CGGAGAACCCATATGTCTTTAGCAAAAGA *5 |
| Pr34 | CitT |  | ATCTACAATTGTTAGTTCCACATGGCGAG *6 |
| *, The underlines indicate restriction sites; *1, *Nco* I; *2, *Xho* I; *3, *Pst* I; *4, *Kpn* I; *5, *Nde* I; *6, *Mfe* I. | | | |
