# Supplementary material for: Generation of Circularly Permuted Fluorescent-Protein-Based Indicators for In Vitro and In Vivo Detection of Citrate
Source: PLoS One. 2013 May 22;8(5):e64597. doi: 10.1371/journal.pone.0064597 (PMC3661591; doi:10.1371/journal.pone.0064597)
Supplement: Table S2 — Metal ion sensitivity of CF98. (DOCX) [file pone.0064597.s002.docx]

| **Table S2 Metal ion sensitivity of CF98** | | |  |  |
| --- | --- | --- | --- | --- |
| Reagent | FI504/FI413 of CF98 | | | |
|  | Citrate 0 mM | 5 mM | | |
| No additive | 7.06 ± 0.235 | 11.7 ± 0.338 | |  |
| MgCl_2_ | 6.92 ± 1.09 | 10.3 ± 0.467 | |  |
| CaCl_2_ | 6.80 ± 0.915 | 10.0 ± 0.310 | |  |
| MnCl_2_ | 6.93 ± 1.61 | 10.1 ± 0.574 | |  |
| FeCl_2_ | 5.67 ± 0.729 | 9.26 ± 1.89 | |  |
| NiCl_2_ | 8.24 ± 1.40 | 6.92 ± 3.62 | |  |
| CuCl_2_ | 3.18 ± 0.925 | 6.54 ± 0.847 | |  |
| ZnCl_2_ | 2.54 ± 0.276 | 9.00 ± 0.810 | |  |
| AgNO_3_ | 6.90 ± 3.47 | 3.76 ± 1.77 | |  |
| HgCl_2_ | 7.44 ± 0.408 | 8.17 ± 0.590 | |  |
| Fluorescence intensities (FIs) were measured at 525 nm when excited at 504 nm (FI504) and 413 nm (FI413) using the indicated reagents containing 50 mM HEPES-NaOH buffer (pH 7.0) with the indicated citrate concentrations (0 mM or 5 mM). The reagents indicated were used as sources of metal ions, and the final concentrations of metal ions were adjusted to 1 mM. The values are averages and standard deviations of three independent experiments. | | | | |
