# Supplementary figures and images for: Generation of Circularly Permuted Fluorescent-Protein-Based Indicators for In Vitro and In Vivo Detection of Citrate
Source: PLoS One. 2013 May 22;8(5):e64597. doi: 10.1371/journal.pone.0064597 (PMC3661591; doi:10.1371/journal.pone.0064597)

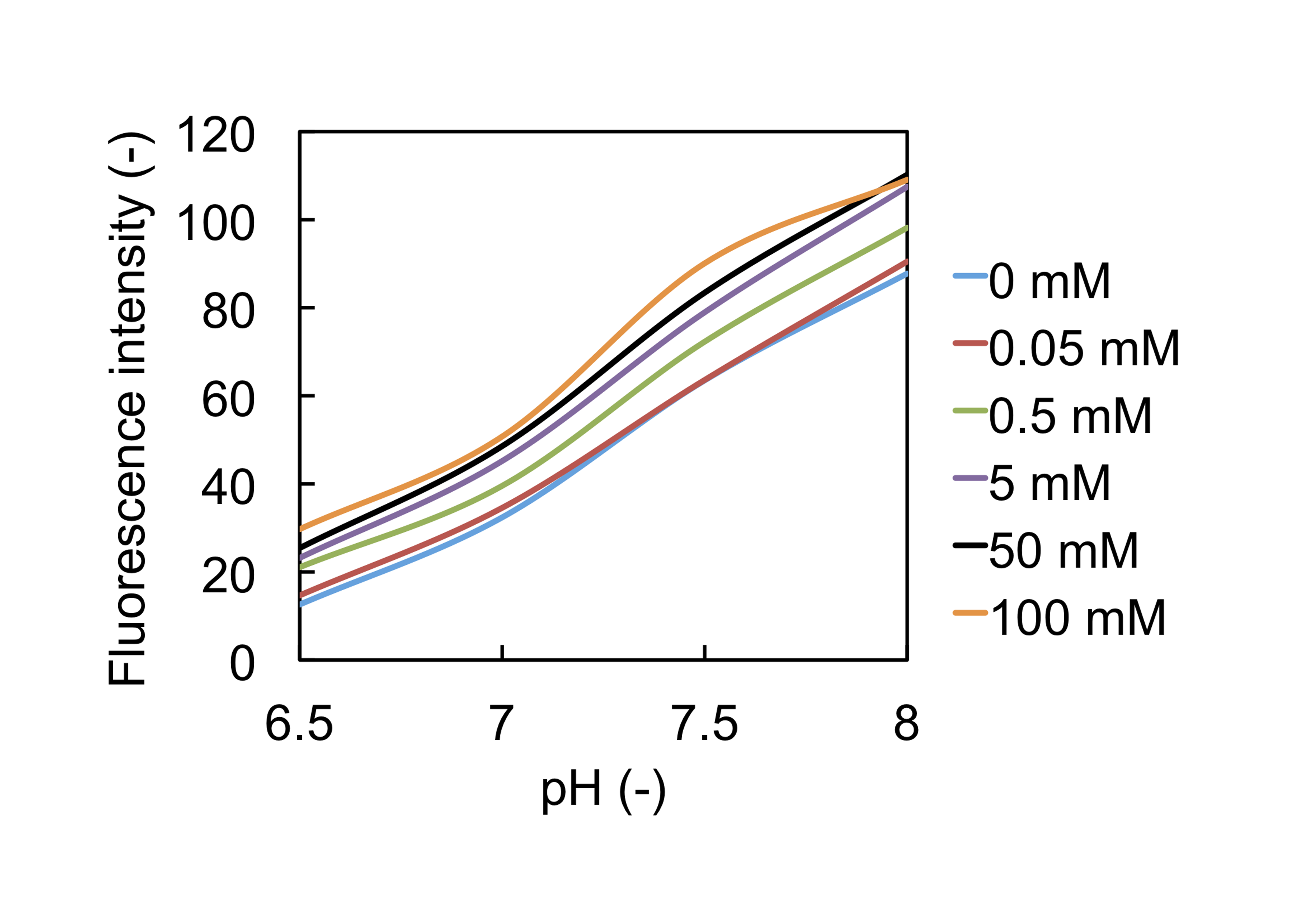

Supplement: Figure S1 — pH sensitivity of CF98. The fluorescence intensities (excitation at 504 nm and emission at 525 nm) of CF98 were measured using a series of buffers prepared with pHs ranging from 6.5 to 8.0 containing the indicated citrate concentrations. CF98 is sensitive to pH, but the magnitude relationship between fluorescence intensity and citrate concentration is maintained. (TIF) [file pone.0064597.s003.tif]

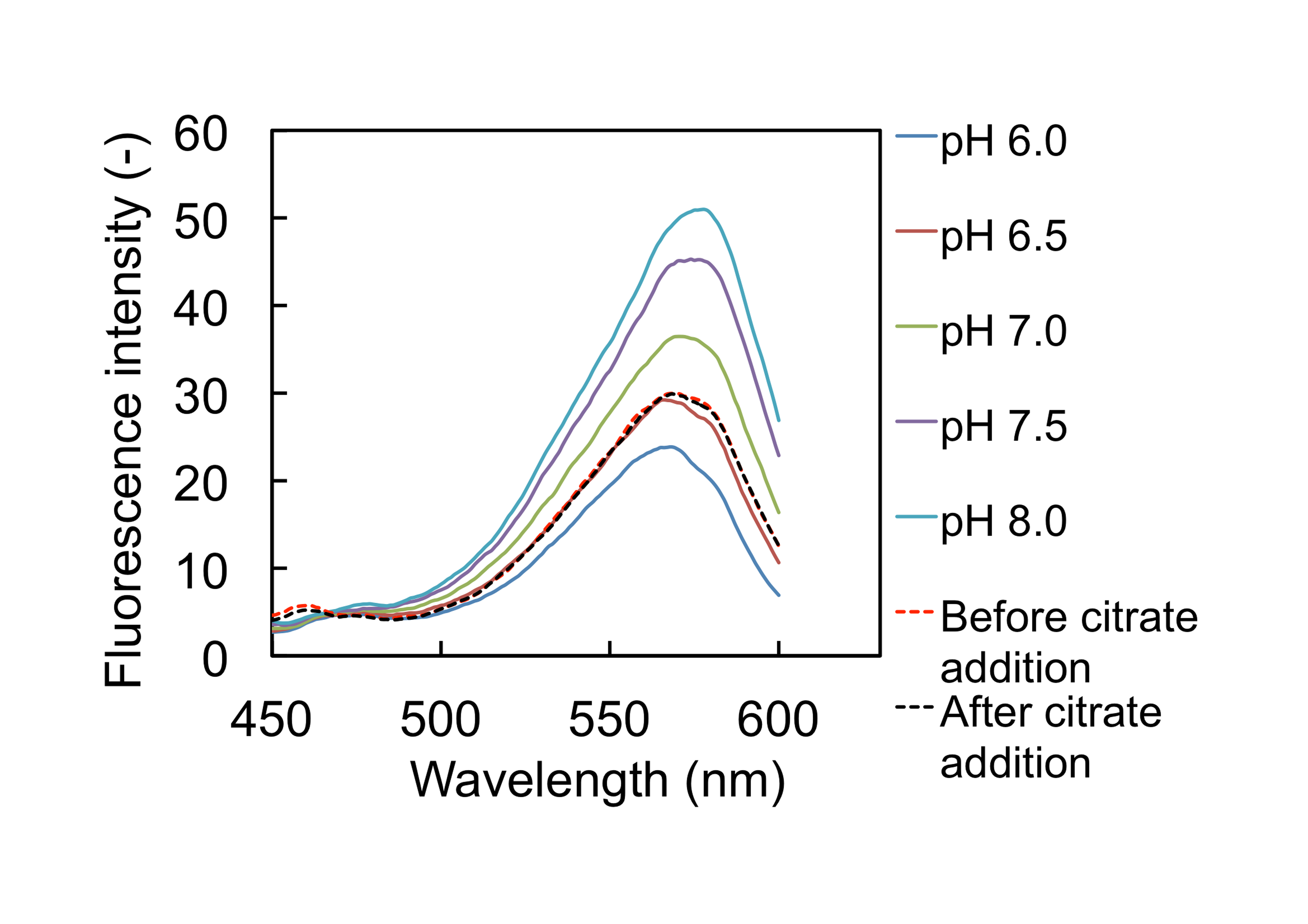

Supplement: Figure S2 — Effects of pH on the excitation spectra from aliquots of the cell suspension of SNARF-5F-loaded E. coli BL21(DE3)/pECF98+pRCITT. The solid lines represent the excitation spectra of SNARF-5F from the cell suspensions diluted with calibration solutions for each of the indicated values of pH. The dotted lines represent the excitation spectra of SNARF-5F from the cell suspensions measured at 50 s after addition of the citrate-containing buffer to give the final concentration of 2.5 mM. Emission at 630 nm. (TIF) [file pone.0064597.s004.tif]

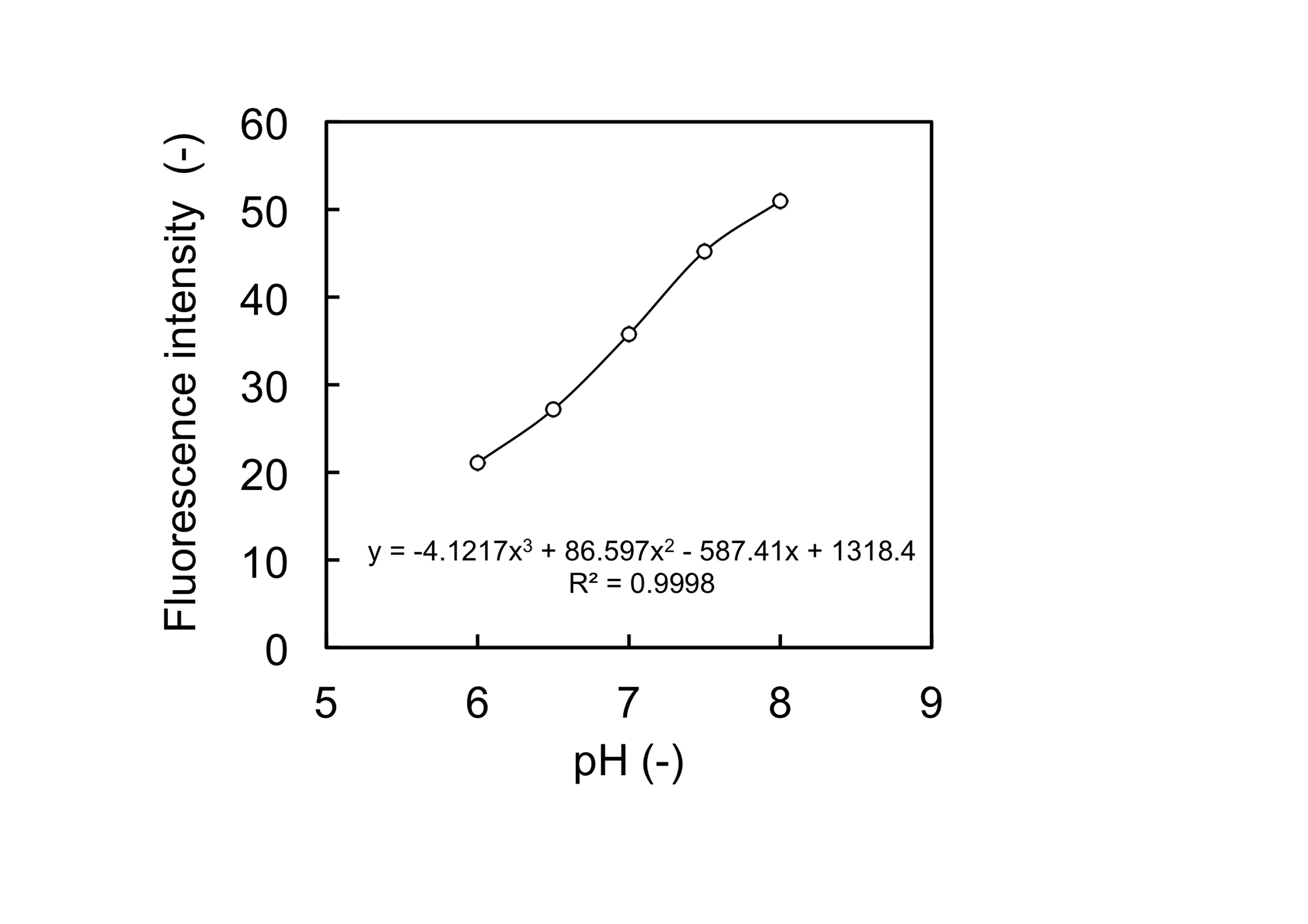

Supplement: Figure S3 — Calibration curve of intracellular pH versus fluorescence intensity of SNARF-5F-loaded E. coli cells. The pH calibration curve constructed by plotting fluorescence intensities (excitation at 577 nm and emission at 630 nm) of SNARF-5F from aliquots of the cell suspension of SNARF-5F-loaded E. coli BL21(DE3)/pECF98+pRCITT diluted with each pH calibration solution against pH. (TIF) [file pone.0064597.s005.tif]

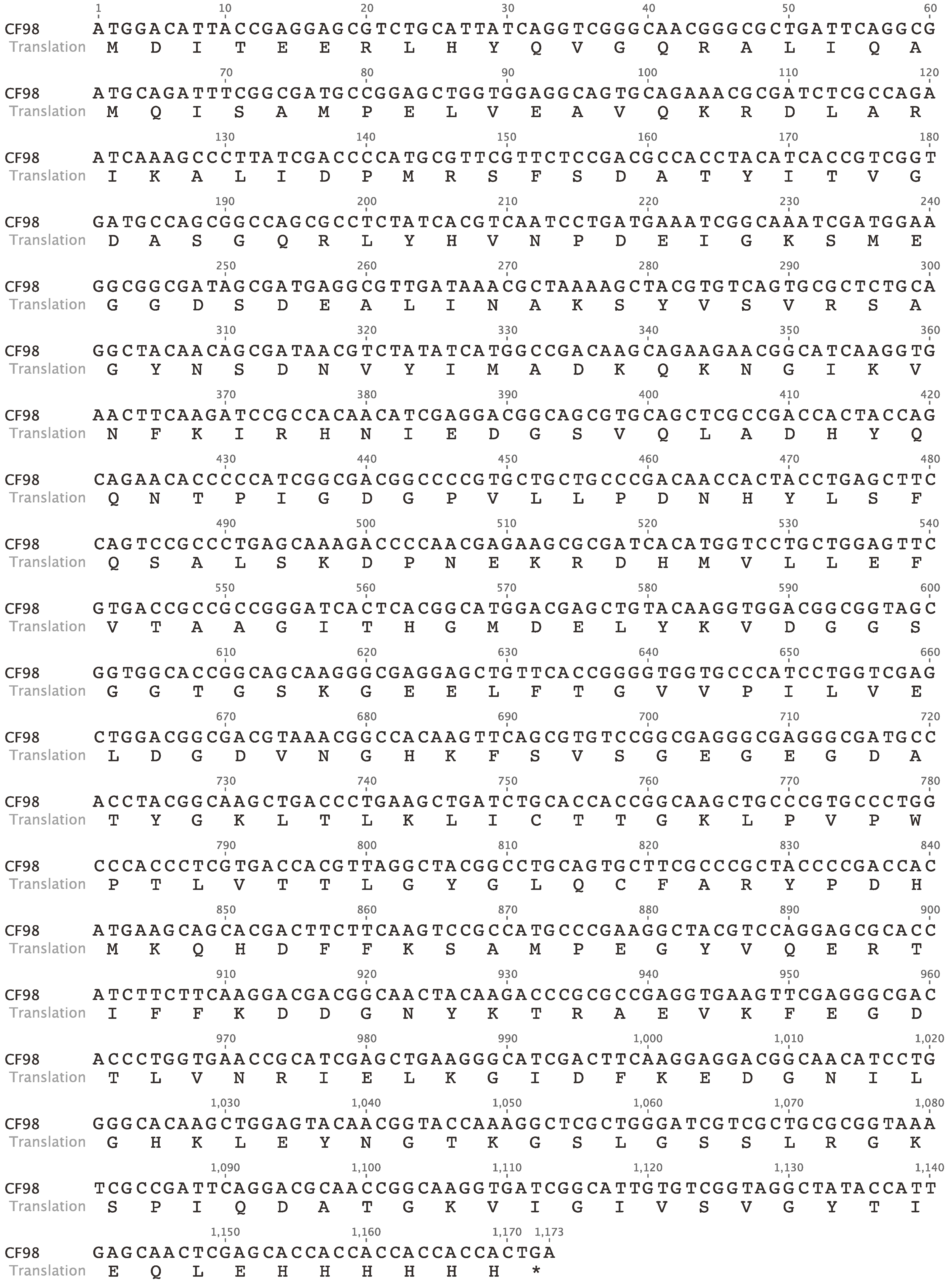

Supplement: Figure S4 — The full nucleotide and protein sequences of CF98. (TIFF) [file pone.0064597.s006.tiff]
